# Highly reversible oxygen redox in layered compounds enabled by surface polyanions

**DOI:** 10.1038/s41467-020-17126-3

**Published:** 2020-07-08

**Authors:** Qing Chen, Yi Pei, Houwen Chen, Yan Song, Liang Zhen, Cheng-Yan Xu, Penghao Xiao, Graeme Henkelman

**Affiliations:** 1 School of Materials Science and Engineering, and MOE Key Laboratory of Micro-Systems and Micro-Structures Manufacturing, Harbin Institute of Technology, Harbin, 150001 China; 2 Department of Chemistry and the Oden Institute for Computational Engineering and Sciences, The University of Texas at Austin, Austin, TX 78712-0165 USA; 3 College of Materials Science and Engineering, Chongqing University, Chongqing, 400044 China; 4 School of Materials Science and Engineering, Harbin Institute of Technology (Weihai), Weihai, 264209 China; 5 School of Materials Science and Engineering, Harbin Institute of Technology (Shenzhen), Shenzhen, 518055 China; 6 Materials Science Division, Lawrence Livermore National Laboratory, Livermore, CA 94550 USA

**Keywords:** Energy, Electronic materials, Batteries

## Abstract

Oxygen-anion redox in lithium-rich layered oxides can boost the capacity of lithium-ion battery cathodes. However, the over-oxidation of oxygen at highly charged states aggravates irreversible structure changes and deteriorates cycle performance. Here, we investigate the mechanism of surface degradation caused by oxygen oxidation and the kinetics of surface reconstruction. Considering Li_2_MnO_3_, we show through density functional theory calculations that a high energy orbital (lO_2*p*’_) at under-coordinated surface oxygen prefers over-oxidation over bulk oxygen, and that surface oxygen release is then kinetically favored during charging. We use a simple strategy of turning under-coordinated surface oxygen into polyanionic (SO_4_)^2−^, and show that these groups stabilize the surface of Li_2_MnO_3_ by depressing gas release and side reactions with the electrolyte. Experimental validation on Li_1.2_Ni_0.2_Mn_0.6_O_2_ shows that sulfur deposition enhances stability of the cathode with 99.0% capacity remaining (194 mA h g^−1^) after 100 cycles at 1 C. Our work reveals a promising surface treatment to address the instability of highly charged layered cathode materials.

## Abstract (web summary)

Oxygen-anion redox in lithium-rich layered oxides can boost the capacity of lithium-ion battery cathodes. Here, the authors investigate the mechanism of surface degradation caused by oxygen oxidation and the kinetics of surface reconstruction.

## Introduction

In the past few decades, Li-ion batteries with high energy density have been intensively investigated due to the increasing requirements of the emerging market of mobile devices and electric vehicles. Cathode materials, including Li-rich NMC (Li_1.2_Ni_0.13_Mn_0.54_Co_0.13_O_2_) with high capacity and energy density, have thus attracted tremendous attention. At present, the capacity of conventional cathode materials is largely restricted by the limited capability of removable Li^+^ ions, which are charge-compensated by cationic redox, so that the practical capacity has approached a limit of about 200 mA h g^−1^. Recently, a new anion redox couple, the reversible O^2−^/(O_2_)^*n*−^ redox reaction, has been widely understood to take place in lithium-rich layered oxides (LLO), which offers a new paradigm to boost the capacity of cathode materials up to ∼300 mA h g^−1^.

The chemical nature of oxygen with redox activity in LLO has been studied theoretically. Based on observations of the local environments of O ions in LLO, it was found that the O-2*p* orbital along the Li–O–Li direction will generate a specific nonbonding band with a higher energy level^1^. These nonbonding orbitals will be oxidized preferentially during the delithiation process and thus contribute to the extra capacity of LLO. It has been further understood that a reversible oxygen redox can be achieved only when the antibonding transition-metal (TM)–O band of TMO_6_ ligands partially overlaps with the O-2*p* nonbonding band or with the antibonding orbital of O–O dimers^2,3^. However, for earth-abundant 3*d*-TM ions, the insufficient covalency of TM–O band prohibits charge transfer between TM and O^2−^, resulting in an undesirable irreversible oxygen oxidation^4,5^ and subsequent structural degradation and detrimental electrochemical performance^6,7^. This chemical picture accounts for the observation of gaseous oxygen evolution in the first cycle by in situ differential electrochemical mass spectrometry^8^. Recently, an alternative redox mechanism in Li-excess manganese oxides was proposed^9^; a small fraction of Mn^4+^ will be oxidized to Mn^7+^ followed by the spontaneous dimerization of oxygen during charge. The multiple redox process causes the voltage hysteresis and voltage fade.

Until now, most theoretical research has focused on the thermodynamics of oxygen redox, and the inevitable thermodynamic instability at highly charged states has been confirmed by calculating the enthalpy of the oxygen loss reaction, especially in 3*d*-TM containing LLOs^10,11^. However, even though the peroxide dimer or a trapped oxygen molecule could form inside the material^9,12,13^, gaseous oxygen release from the bulk is kinetically unlikely^14,15^, due to kinetic prohibition for oxygen interstitial migration^16^. This explains previous experiment results showing that the oxygen in bulk LLO can undergo reversible redox and contribute to the reversible capacity^17–20^. Therefore, the kinetics of oxygen loss is essential to understand the irreversible capacity loss during cycling in LLO. To optimize the electrochemical performance of LLOs, it is critical to mitigate surface oxygen release and thus kinetically impede oxygen loss in the entire material while not affecting oxygen redox in the bulk material. As long as O is retained in the structure, other structural changes are likely reversible upon discharging^21^. Various surface modifications have therefore been proposed to suppress the irreversible process, and substantial improvements have been achieved^22–26^. Nevertheless, a fundamental understanding of surface oxygen evolution, including energetics and kinetics, is still missing, and this is essential to understanding the reversibility of anion redox and the metastable nature of highly charged layered materials.

In this work, we theoretically investigate the surface oxygen on Li_2_MnO_3_, which is the end member and primary component after the “activation” of LLO. The overoxidation of surface oxygen is attributed to its undercoordinated configuration with cationic vacancies on the surface, which creates an additional higher energy orbital (lO_2*p*’_). The calculated minimum energy path (MEP) shows that the kinetically favored surface oxygen release gives rise to surface Mn back diffusion and thus triggers a spinel-like phase transformation. We therefore induce surface polyanion formation by sulfur deposition to avoid the nonbonding coordination of surface oxygen and thus stabilize the surface. These theoretical predictions are confirmed by experimental cycling tests carried out in a practical LLO, Li_1.2_Ni_0.2_Mn_0.6_O_2_. This study uncovers a promising path toward addressing the issue of cycled structure instability in current cathode materials that resulted from surface anionic oxidation.

## Results

### Surface oxygen oxidation and correlation with surface structural transformation in Li_2_MnO_3_

**Fig. 1:**
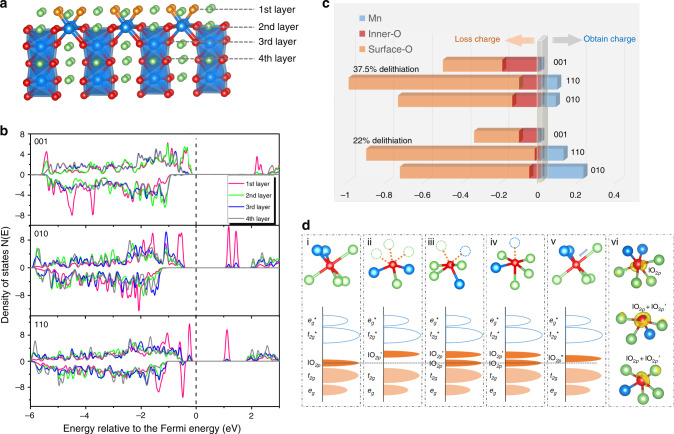
Redox mechanism on the surface of Li_2_MnO_3_. **a** Crystal structure of Li_2_MnO_3_ (010) surface. The oxygen ions are divided into four layers from the outmost surface to the inner; each layer has the same number of O ions. The blue, green, and red/golden balls represent Mn, Li, and O ions, respectively. **b** Calculated LDOS of oxygen ions from the first layer to the fourth layer in the (001), (010), and (110) surfaces, respectively. The Fermi level is indicated by the dashed line. **c** Bader analysis of the surface O, inner O, and Mn ions of the three slabs in different delithiation states. Negative values of the change of Bader charge indicate electron loss of the ions. **d** Schematic diagram of various local atomic configurations, as well as the electronic structures and the calculated ELF. i, O_int_, ii, O_sur_ on the (001) surface, iii, O_sur_ on the (010) surface and partial O_sur_ on the (110) surface, iv, partial O_sur_ on the (110) surface, v, oxygen ions in the second layer of the (001) surface, and vi, ELF values computed (isovalues of 0.7) for O_int_ and O_sur_ in iii and iv. The hollow blue area, filled orange area, and filled light-orange area in each electronic state represents the empty antibonding (M–O)^*^ band, O(2*p*) lone-pair band, and bonding (M–O) band, respectively.

To study the surface oxygen evolution, three low-index surfaces of Li_2_MnO_3_, i.e., (001), (010), and (110), were examined. These surfaces are considered to be stable and electrochemically active^27^. Various terminations and their corresponding surface Li-binding energies were examined (Supplementary Fig. 1), revealing that at the beginning of charge, the most stable (001) surface is terminated by O ions, and the stable (010)/(110) surfaces are terminated by Li ions. To distinguish the oxygen oxidation in surface and bulk, we studied O ions at different layers from the outermost layer to the innermost, as shown for the (010) surface in Fig. 1a (the other two surfaces are shown in Supplementary Fig. 2). The redox activity of oxygen atoms from the first layer to the fourth layer in the three surfaces was predicted by the calculated local density of states (LDOS) using the HSE06 hybrid functional^28^ (Fig. 1b). It is shown that the bands near the Fermi level are mostly from the oxygen ions in the outermost layer. Here, the O ions in the first layer are denoted as O_sur_, while in the other layers, are considered as O_int_. It can then be concluded that the 2*p* orbital of O_sur_ has a higher energy level than that of O_int_, suggesting a preferred oxidation of O_sur_ in the delithiation process.

Prior to investigating oxygen reactions on the surface, the electrochemical activity of each surface was examined by evaluating its Li transfer ability. We calculated the preferred diffusion paths of Li extraction from each surface and the corresponding kinetic barriers (Supplementary Fig. 3), showing that the rate-limiting mechanisms of sequential diffusion steps are similar, and thus the three surfaces can delithiate spontaneously. To simulate a practical degree of O^2−^ oxidation in Li-rich layered oxides (0.7–0.8 e^−^ per formula unit)^8,29^, the delithiated state on each surface was set as 37.5% (0.75 e^−^ formally extracted from O per formula unit). The basin-hopping global optimization algorithm was then utilized to search for the lowest energy structure in each surface, to find that Li vacancies are primarily distributed in the outermost part of the slab (Supplementary Fig. 4). This is in agreement with a previous observation from electron energy loss spectroscopy (EELS), which indicated that the concentration of Li in the surface is less than that in the interior area, even when the cell is fully discharged^30^. As a result of the inhomogeneous distribution of Li vacancies, three pairs of O_sur_ rotate to spontaneously form O_sur_–O_sur_ dimers on the (010) and (110) surfaces at the 37.5% delithiation state, whereas no O_int_–O_int_ formation is seen in the slabs, indicating a higher oxidation state of O_sur_. For the (001) surface, the O–O dimers also form only at the surface with a low barrier (0.2 eV), consistent with a higher oxidation state of O_sur_; details of this mechanism will be discussed later.

To quantify the charge state of O_sur_ and O_int_ during delithiation, we calculated the Bader charge (per atom) of O and Mn atoms in each surface at two delithiated states (Fig. 1c). A negative value for the change of Bader charge indicates electron loss at the ions, corresponding to the oxidation reaction. As revealed by the Bader analysis, charge compensation of the whole system is dominated by the oxidation of O_sur_ with more electron loss, although the oxidation of O_int_ is enhanced with the state of delithiation from around 22% to 37.5%; the slight reduction of Mn suggests charge transfer between O and Mn. The higher oxidation of O_sur_ than O_int_ during delithiation is not surprising because the Li vacancies are prone to aggregation at the surface, which requires more charge compensation.

In essence, the prior oxidation of O_sur_ can be understood by the specific surface-coordinated configuration at the electronic structure level. In contrast with O_int_ that has one lO_2*p*_ pair (an O(2*p*) lone pair)^31^ (i in Fig. 1d), the local coordination of O_sur_ in our models can be used to categorize O_sur_ into three species with different cationic vacancies (ii, iii, and iv in Fig. 1d). These surface cationic vacancies result in a reduced bonding of O_sur_ along the $\square$-O_sur_-Li direction ($\square$ represents cationic vacancies) with respect to that of lO_2*p*_ in O_int_. The $\square$-O_sur_-Li configuration on the surface therefore generates an lO_2*p*_’ orbital with higher energy level than lO_2*p*_, which can be seen in the higher energy level of O in the first layer, as shown in the LDOS (Fig. 1b). The increased number of lone-pair orbitals (iii and iv in Fig. 1d) can been directly observed in the electron localization function (ELF)^31,32^ results (vi in Fig. 1d). It should be mentioned that the oxygen ions in the second layer of (001) surface have a comparable energy level with that of the first layer, which is probably a result of the elongated Li–O distance in the second layer that raises the energy level of lO_2*p*_ to lO_2*p*_” (v in Fig.1d).

**Fig. 2:**
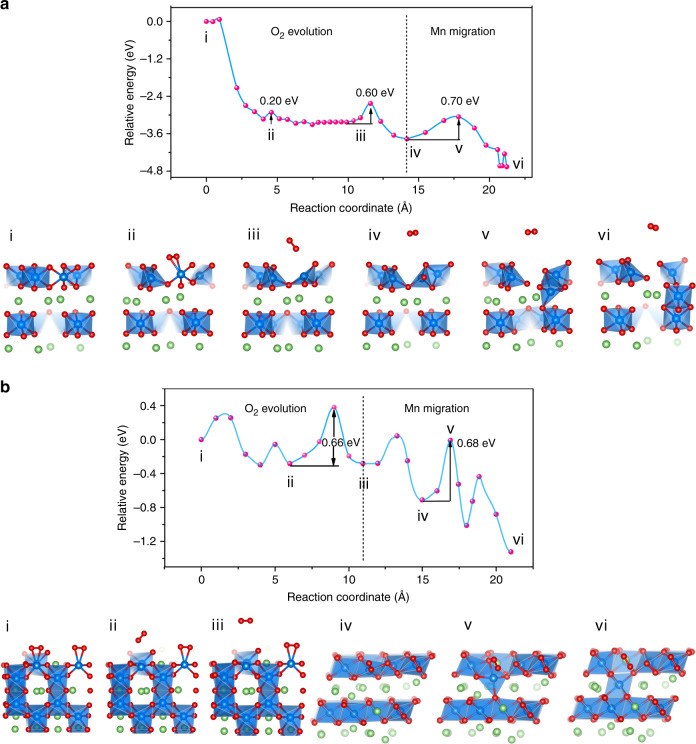
Minimum energy path (MEP) plot for O_2_ evolution and Mn migration at the surface of Li_2_MnO_3_. **a**, **b** MEP plot for O_2_ evolution and Mn migration from the TM layer to the Li layer (upper) in the (001) (**a**) and (010) (**b**) surfaces, and the corresponding structures of some intermediates and transition states (below). All slabs in (**a**) and the first three slabs in (**b**) are shown as side views, while the last three slabs in (**b**) are top views to better visualize the Mn migration pathway.

The kinetic mechanism of irreversible oxygen release and the subsequent structural transformation were investigated to directly correlate the oxygen oxidation with O_2_ gas release and surface reconstruction. We calculated the minimum energy path (MEP) of the structural evolution on the (001) and (010) surfaces upon 37.5% delithiation (Fig. 2). The kinetic barriers of irreversible O_2_ release from the (001) and (010) surfaces are 0.60 and 0.66 eV, respectively, implying that the reaction of O_2_ released from the crystal is kinetically feasible at room temperature. Here, the concentration of oxygen vacancies after O_2_ evolution in the first layer is 33% in the (001) slab and 25% in the (010) slab. The onset of O_2_ evolution at these surfaces is expected with activation barriers no greater than 0.60 and 0.66 eV. The surface O_2_ release barrier will increase with the presence of oxygen vacancies and Mn densification near the surface, which explains why the irreversible oxygen release occurs primarily during the first charging cycle. We also utilized strongly constrained and appropriately normed density functional (SCAN)^33,34^, which is more accurate for the calculation of gas-phase oxygen, to obtain the MEP for lattice oxygen evolution to O_2_ on both surfaces (Supplementary Fig. 5), further revealing the kinetically feasible barriers (<0.60 eV) for O_2_ release. As mentioned previously, the facile oxidation of O_sur_ in Li_2_MnO_3_ is due to its reduced bonding coordination. Therefore, it should be possible to increase the O_2_ evolution barrier to mitigate or delay the irreversible O_2_ release by introducing electron donor.

With the surface oxygen dimer separated from Mn, the MnO_6_ octahedron changes to MnO_4_ tetrahedron and the Mn moves to a more stable environment. From the calculated MEP plot of Mn migration followed by oxygen release on both surfaces (Fig. 2), the final states of the MnO_6_ octahedron located in the Li layer are more stable than the initial states of the MnO_4_ tetrahedron in the TM layer. During the process of Mn migration, a regular MnO_4_ tetrahedron is formed spontaneously (on the (001) surface) or with a small barrier (on the (010) surface) after oxygen release, while the subsequent migration of Mn ions into nearby interstitial octahedral sites in the Li layer is a slow step with a barrier of 0.7 eV. Meanwhile, Mn migration promotes the rearrangement of Li ions from interstitial octahedral sites to tetrahedral sites, thus forming a local spinel-like structure. The calculated surface kinetic process provides theoretical support for the experimentally observed structural transformation from a layered to spinel-like or rock-salt phase in cycled LLO. In addition, the high reverse barrier for Mn migration back to the initial position during discharge accounts for the deterioration of electrochemical reversibility.

From the above discussion, the specific coordination configuration of O_sur_ changes its electronic structure by introducing an additional lO_2*p*’_ band with a higher energy level than lO_2*p*_, inducing the preferred oxidation of O_sur_. In addition, the calculated kinetic barrier of O_sur_ release (≤0.66 eV) is accessible at room temperature. The kinetically feasible oxygen release then leads to a surface structural reconstruction via Mn migration. At this point, the reverse process in the discharged state, which requires O_2_ dissociation and Mn extraction, becomes kinetically prohibitive. We did not calculate the barrier for the reverse process, but it is well known that O_2_ dissociation on oxides is kinetically hindered at room temperature^35,36^. Consequently, O_2_ release is the detrimental factor for reversibility. Strategies that target manipulation of the electronic structure of O_sur_ and O_2_ evolution barrier are thus promising routes to mitigate O_2_ release and retain the reversibility of layered compounds.

### Surface polyanion induction effect stabilizes surface oxygen

Since the overoxidation of O_sur_ stems from a deficiency of cationic bonding on the surface, we aim to reintroduce cationic species into the surface to bond with O_sur_, with the expectation of reducing nonbonding coordination. Inspired by the achievement of stabilizing oxygen through the inductive effect in polyanion compounds^37^, surface deposition of less electronegative sulfur was utilized to stabilize O_sur_ in Li_2_MnO_3_. Sulfur deposition was carried out on three surfaces of Li_2_MnO_3_. The deposition energy at different sites and with different sulfur concentrations is compared in Supplementary Fig. 6. The results show that sulfite-like species (SO_3_)^*x*−^ form on the (001) surface and (SO_2_)^*y*−^ forms on the (010) and (110) surfaces; these surface oxysulfides were confirmed to be thermodynamically stable. The formation of (SO_*n*_)^*m*−^ polyanions suggests that electrons transfer from the less electronegative sulfur to O_sur_ by the inductive effect. Li-ion mobility in the sulfur-deposited structure was evaluated by calculating the preferred diffusion paths and the migration barriers (Supplementary Fig. 7). The calculated diffusion barriers (between 0.71 and 0.86 eV) in all the three surfaces are comparable with those before sulfur deposition (between 0.74 and 0.79 eV), indicating that the sulfur deposition does not hinder transport of Li from the surface.

**Fig. 3:**
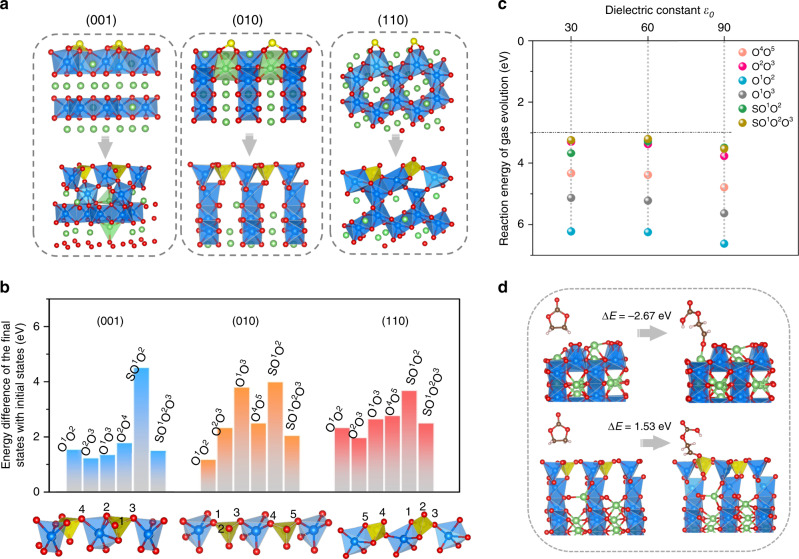
The surface structures of Li_2_MnO_3_ with sulfur deposition and the stability of surface O after delithiation. **a** Structures of the (001), (010), (110) surfaces with sulfur deposition (upper) and the reconstructed configurations upon 37.5% delithiation (below). The yellow spheres represent S ions. **b** Energy required for gas evolution on the three delithiated surfaces. Each exposed O atom on the surface is labeled with a different number and then is grouped together or with S atom to form the potential gas products, such as O_2_, SO_2_, or SO_3_. Each slab with gas evolution to the vacuum is taken as the final state. **c** Reaction energy of (010) surface oxygen evolution in implicit solvents with different dielectric constants. **d** EC molecular adsorption on delithiated (010) surface before and after surface treatment and ring opening of cyclic carbonate.

After extraction of 37.5% Li, no O–O dimers are observed on the sulfur-deposited surfaces; instead, the (SO_3_)^*x*−^ and (SO_2_)^*y*−^ species are oxidized to sulfate species (SO_4_)^2−^ with tetrahedral configurations (Fig. 3a). This indicates that the redox center on the sulfur-deposited surfaces has shifted from O^2−^/O^−^ to (SO_*n*_)^*m*−^/(SO_4_)^2−^, that is, sulfur ions provide the charge compensation on the surface in the first delithiation step. The top view of each surface after 37.5% delithiation (Supplementary Fig. 8) shows that all the O_sur_ has been coordinated with sulfide ions, indicating that the undesirable nonbonding coordination of O_sur_ can be effectively avoided by sulfur deposition. Note that the two outermost Mn ions on the (001) surface spontaneously migrate into Li-vacancy sites in the Li layer to form MnO_6_ octahedra after Li extraction, while no Mn migration was observed on the (010) and (110) surfaces. This can be attributed to the different orientations of the repulsive interaction between the high-valency S and Mn ions in the different surfaces. Nevertheless, the reconstructed surfaces reveal that sulfur deposition will likely promote the formation of Li/Mn antisite mixing during delithiation. The delithiation voltages of the three structures (Fig. 3a) are calculated to be 2.97, 3.05, and 2.86 eV, respectively. These low voltages suggest that the delithiated states are stable, i.e., there is facile formation of the reconstructed surfaces.

To determine the stability of O_sur_ after sulfur deposition during charging, we calculated the energetics of all possible oxygen evolution mechanisms on all three surfaces, to release O_2_, SO_2_, or SO_3_. To simplify the description of our results, we labeled the O_sur_ in each surface, and assigned it to various possible products in the gas phase (O_2,_ SO_2_, and SO_3_) as the final states (see Fig. 3b). The energies of these final states are at least 1.2 eV higher than the initial states, indicating that O_sur_ in the form of polyanions are energetically stable against gas evolution. We then utilized the more accurate SCAN functional to calculate the energy difference on (001) surface, to spot-check the possibility of oxygen loss. The calculated energy differences are all greater than 2.8 eV, showing even higher stability than the PBE + U results. Surface oxygen stabilization is expected to enhance the reversible oxygen redox and promote capacity retention.

In addition, we consider the (010) surface as representative to check the surface stability under more practical electrolyte conditions. The gas evolution behavior in the presence of solvent was investigated using an implicit solvent model as implemented in the VASPsol package^38^. The reaction energy of all possible O_sur_ evolution with solvent dielectric constants of 30, 60, and 90 (*ε*_0_ = 2 in dimethyl carbonate (DMC), *ε*_0_ = 90 in ethylene carbonate (EC))^39^ is calculated to be greater than 3.0 eV (Fig. 3c), suggesting that the polyanionic (SO_4_)^2−^ is stable with or without the solvation effect. Reaction with the electrolyte was also investigated. Referring to previous reports on the reaction between Li transition-metal oxides and conventional electrolytes, EC has been found to preferentially adsorb and react with the electrodes^40,41^. In general, EC reaction/decomposition initiates by breaking one of the two C–O bond in the ring on the cathode surfaces^42,43^, and finally evolves to CO_2_ and various other organic species^44,45^. Therefore, our calculations focus on the first step of EC decomposition, the ring opening of cyclic carbonate after EC molecular adsorption on the delithiated (010) surface, as shown in Fig. 3d. The reaction energy was calculated to be −2.67 on the pristine surface and 1.53 eV on the S-deposited surface using the SCAN functional, indicating that the onset of EC degradation is exothermic on the S-free surface of delithiated Li_2_MnO_3_, but energetically prohibitive after forming the polyanionic configuration. This is in agreement with previous reports that surface oxygen of charged layered oxides can attack carbonate solvents and catalyze electrolyte decomposition^42,46^. It can therefore be concluded that the polyanionic (SO_4_)^2−^ species could stabilize the surface of Li_2_MnO_3_ not only by preventing gas releasing in solution, but also by inhibiting reactions with the electrolyte. The mechanism of prevention of polyanionic (SO_4_)^2−^ against reaction with EC molecule is analyzed below.

**Fig. 4:**
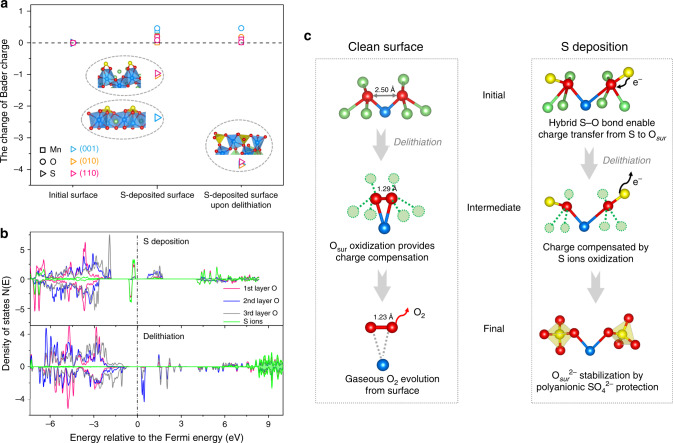
Surface charge transfer of sulfur-deposited Li_2_MnO_3_ and the mechanism of surface O stabilization. **a** The change in Bader charge of surface Mn, O, and S (per atom) on the three outermost surfaces during the process of S deposition and latter extraction of 37.5% Li. Bader charge of surface Mn, O (per atom) on the initial surface, and S atoms has been normalized to zero. **b** Calculated local density of states of the first layer O to the third layer O and S ions upon S deposition and 37.5% delithiation. Here the (010) surface was taken as characteristic of the three surfaces of Li_2_MnO_3_. **c** Schematic of how S deposition stabilizes surface O during delithiation in the first cycle (right), by contrast to O_2_ evolution from the clean surface (left). From top to bottom: the coordination environment of the surface O ions pairs in full lithiation; the intermediate local structure after 37.5% delithiation; the final stable configuration of surface O ions pairs after 37.5% delithiation.

To clarify the electronic structure of S deposition, we analyzed the Bader charge during the process of deposition and delithiation, and calculated the LDOS using the HSE06 hybrid functional (Fig. 4a, b). The Bader analysis shows an obvious oxidation of S ions and a slight reduction of O_sur_ upon sulfur deposition (Fig. 4a). This inductive effect with charge transfer from S to O_sur_ facilitates the formation of oxysulfide (SO_*n*_)^*m*−^ species. After extraction of 37.5% Li ions, the (SO_*n*_)^*m*−^ species are further oxidized to polyanionic (SO_4_)^2−^, whereas the oxidation states of O_sur_ and Mn ions are barely changed, demonstrating that S ions, instead of O_sur_, provide the charge compensation on the surface in the first delithiation process. The calculated LDOS on the (010) surface shows that the orbital of the deposited S ions is hybridized with the first layer of O ions and lies right below the Fermi level (Fig. 4b). The high energy level of the hybridized S–O orbital suggests a preferential oxidation of (SO_*n*_)^*m*−^ species. In the 37.5% delithiated state, with S ion loss of electrons, these bonding S–O states move down to −7 eV, and the antibonding S–O states move to 9 eV above the Fermi level, signifying that the polyanionic (SO_4_)^2−^ is redox-inactive during further delithiation or subsequent electrochemical cycles. The more ionic character of the S–O bond in (SO_4_)^2−^ accounts for the large energy difference between the occupied and empty states, thus maintaining surface stability in an electrolyte environment (Fig. 3).

Figure 4c illustrates the mechanism of oxygen evolution and O_sur_ stabilization by polyanion induction. For the clean surface of LLO, O_sur_ release during delithiation is known to arise from its reduced bonding-induced overoxidation, by way of rotation to an O–O dimer, based on the requirement of charge compensation (left part of Fig. 4c). While for the deposited sulfur, the hybrid S–O bond enables charge transfer from S to O_sur_, thus preventing the oxidation and rotation of O_sur_ to form O–O dimers (right part of Fig. 4c). The sulfur ions, which transform from oxysulfide (SO_*n*_)^*m*−^ to the polyanionic (SO_4_)^2−^ species, provide the surface charge compensation in the first charging process, and also maintain redox stable during the following cycles. The strategy to replace the nonbonding coordination of O_sur_ with surface polyanion configuration is not limited to the LLO series of compounds, but to all materials that suffer from surface instability involving O_sur_ oxidation.

### Experimental verification

Sulfide deposition experiments were subsequently conducted to test our computational predictions. Specifically, sulfide deposition on Li_1.2_Ni_0.2_Mn_0.6_O_2_ was conducted and characterized as described in the Supplementary Information. The pristine Li_1.2_Ni_0.2_Mn_0.6_O_2_ and sulfide-deposited samples are labeled as LP, S-50, S-200, and S-500 according to the deposition concentration of S. The morphology and XRD pattern of LP, S-50, S-200, and S-500, as shown in Supplementary Figs. 9 and  10, suggest that the morphology and phase constitution of Li_1.2_Ni_0.2_Mn_0.6_O_2_ powders are unchanged upon S deposition.

**Fig. 5:**
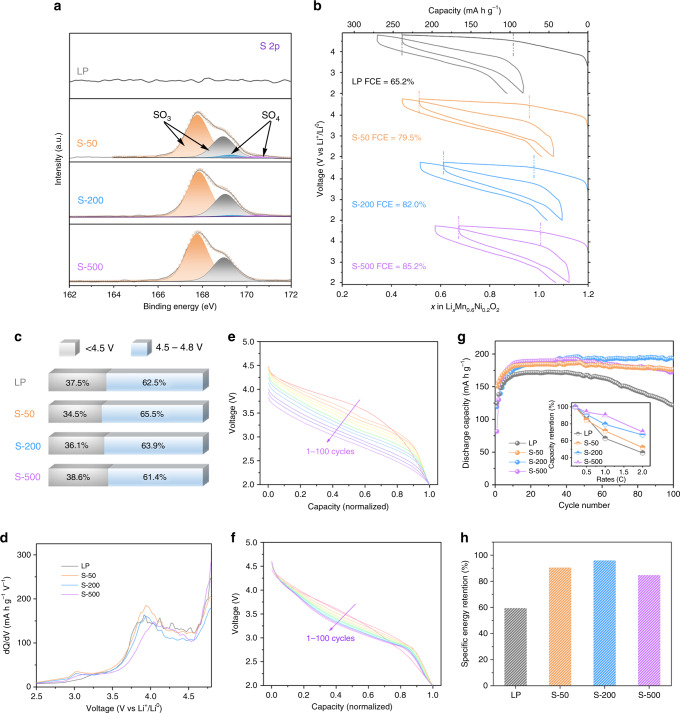
The surface states and electrochemical behavior of products before and after S deposition. **a** S 2*p* spectra of products before and after S deposition. **b** Voltage profiles of the first two cycles obtained at 0.5 C. Regions from 4.5 to 4.8 V are marked with vertical dash lines. **c** Percentage-stacked bar chart of charge capacities in the first cycle obtained at 0.5 C; the total capacities are divided into two sections: <4.5 V and 4.5–4.8 V. **d** dQ/dV curves of the charge process in the second cycle obtained at 0.5 C. **e** Normalized galvanostatic discharge profiles of LP obtained at 1 C. **f** Normalized galvanostatic discharge profiles of S-200 obtained at 1 C. **g** Cyclic performance at 1 C together with the capacity retention at different rates (insets). **h** Specific energy-density retention after 100 cycles at 1 C.

XPS measurements were performed to probe the oxidation states on the surface of each sample (Fig. 5a). The main peaks were observed near 167.8 eV in the S 2*p* core spectra of the S-deposited samples, suggesting the formation of SO_*x*_ species instead of S^2−^ or elemental S^47^. In addition, although SO_3_^2−^ was found to be the major component, SO_4_^2−^ groups with a lower S electron density were observed in S-50 and decreased with a higher concentration of S vapor. This phenomenon is expected to be associated with Li and oxygen vacancies on the surface of LLOs, and will be explored in further work. The O 1s core spectra are shown in Supplementary Fig. 11, in which the deposition of sulfur alters the oxidation state of oxygen by increasing the content of oxygen from SO_*x*_ species, in peaks located near 532 eV^48^.

The charge–discharge curves performed on Li/LP cells (Fig. 5b) are compared with that of S-deposited samples. LP presented the charge capacity of 238 mA h g^−1^ in the first cycle accompanied with a first Coulombic efficiency (FCE) of 65.2%. S absorption showed a slight decreased charge capacity, but an increase in FCE (85% for S-500) in the first cycle; considering that the irreversible processes are closely related to the release of oxygen^29,49–51^, the increased FCE in the S-absorbed samples is likely associated with suppressed irreversible oxygen release from the bulk lattice. It is worth mentioning that previous attempts to increase the FCE of LLO generally shorten the plateau length of the oxygen oxidation reaction (at around 4.5 V), that is, inhibiting the reversible and irreversible oxygen reaction in the first charge^52,53^. The oxygen reaction plateau lengths in this research are compared (Fig. 5c); the slightly higher percentages of charge capacity between 4.5 and 4.8 V in the S-deposited sample (S-50 and S-200) suggest that the oxygen redox reaction is maintained. Thus, it can be deduced that only the irreversible oxygen evolution in LLO was suppressed after S deposition. In addition, the d*Q*/d*V* curves of the second charge shown in Fig. 5d have enhanced redox activity around 3.0 V in the S-deposited samples, indicating the formation of a spinel phase after the first cycle. Considering the fact that traditional LLO → spinel-phase transitions are fairly slow in the first several cycles^54,55^, the spinel phase in the S-deposited samples seems to be more likely initiated by the Li/Mn antisite mixing that is driven by the formation of SO_*x*_.

The normalized galvanostatic discharge profiles of LP and S-200 obtained at 1 C (Fig. 5e, f) are indicative of the suppressed voltage decay in S-deposited samples. As the voltage decay in LLO is induced by the migration of Mn through oxygen vacancies^50,56^, the suppressed voltage decay in S-200 benefits from the inhibition of irreversible oxygen evolution. The cyclic performance at a 1-C rate and the capacity retention at different rates in Fig. 5g show the enhanced electrochemical stability and rate performance of the S-deposited samples. Specifically, the S-200 sample with the best cyclic stability possesses a capacity retention of 99.0% (194 mA h g^−1^) after 100 cycles at 1 C (1 C = 200 mA g^−1^), while the capacity retention is only 70.4% (121 mA h g^−1^) for untreated LP. The specific energy-density retention (Fig. 5h), which is the primary bottleneck of LLO that is influenced by the effects of both voltage and capacity decay, has been significantly improved from 59.5% to 96.0% with an optimal amount of S deposition.

**Fig. 6:**
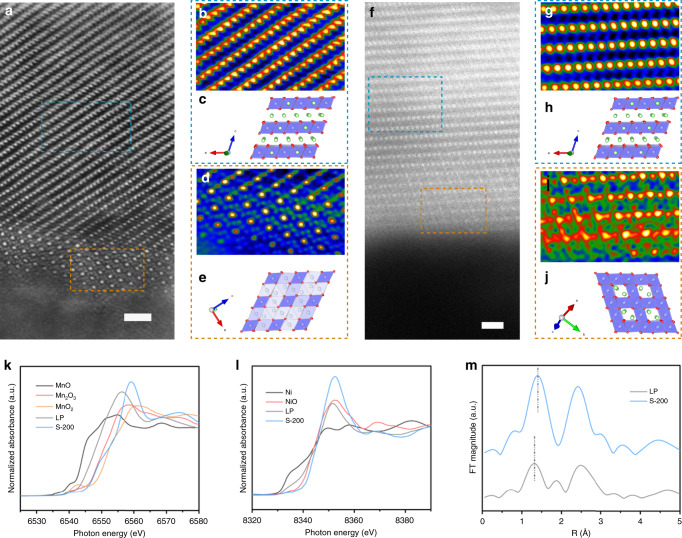
The atomic structural evolution and oxidation-state change of samples before and after cycling. **a**–**e** Atomic configuration of the S-200 before cycling. The overview of HAADF-STEM image (**a**) shows the crystal structure from surface to the bulk of S-200; the blue and orange dash square showed two regions with different atomic configurations. The interior region marked by the blue dashed square in (**a**) has been FFT-filtered and shown in (**b**), and the corresponding atomic model is shown in (**c**). The spots with higher contrast in (**b**) show TM ions with higher Z than Li ions; the Li, M, and O ions have been shown as green, blue, and red spheres in (**c**), respectively. The surface region with cation-mixed Mn_3_O_4_ feature marked by orange dash square in (**a**) has been FFT-filtered and shown in (**d**), and the corresponding atomic model has been drawn (**e**)**. f**–**j** The atomic configuration of S-200 electrode after 100 cycles. The HAADF-STEM image from the surface to the bulk of charged S-200 (**f**) shows the different atomic configurations on the surface area; the blue and orange dashed square shows two regions with different atomic configurations. The layered feature region marked by the blue dashed square in (**f**) has been FFT-filtered as shown in (**g**), and the corresponding atomic model is shown in (**h**). The region with a spinel feature that is marked by an orange dashed square in (**f**) has been FFT-filtered and shown in (**i**), and the corresponding atomic model is shown in (**j**). The scale bar donates 1 nm. **k** Normalized Mn K-edge XANES spectra of cycled LP and S-200. The valence states of Mn were determined by comparison with the spectra of pure-phase MnO, Mn_2_O_3_, and MnO_2_. **l** Normalized Ni K-edge XANES spectra of cycled LP and S-200. The valence states of Ni were determined by comparison against the spectra of pure-phase Ni and NiO. **m** The *k*^3^-weighted Fourier transform magnitudes of Mn K-edge EXAFS spectra obtained from cycled LP and S-200.

The high-angle annular dark-field scanning transmission electron microscopy (HAADF-STEM) technique was used to reveal the atomic structure evolution during de-/lithiation. Prior to electrochemical de-/lithiation, LP showed a typical layered structure on both the interior and the surface region (Supplementary Fig. 12a–c), while the surface of S-200 (2–3 nm) has a different atomic configuration than that of the interior area (Fig. 6a–e). Compared with the layered features from the projection of the <010> zone axis of the *C*2*/m* structure in the interior area (Fig. 6b), TM ions were detected in both the tetrahedral and octahedral sites of the Li layer (O_Li_) near the surface (Fig. 6d)^50,57,58^. Such a surface structure, as presented in the atomic models (Fig. 6e), could be considered as a cation-mixed Mn_3_O_4_-type structure^59–62^, which is likely due to S-deposition-induced Li leaching and subsequent TM ion migration, as predicted by our DFT calculations. The lowered delithiation voltage (by 1.6 V as compared with the bulk^15^) in the S-deposited surface suggests that Li ions are easily leached out from the surface during the heat treatment. The structural change after partial delithiation (Fig. 3a) clearly shows Mn migration to the Li layer. Therefore, although only O ions in the outmost surface will bond with S to form SO_*x*_^2−^ species, based on our DFT calculations, the S-deposition-induced structural evolution could expand into the near-surface region (2–3 nm). After 100 cycles, 2–3-nm-thick surface reconstruction layer with a different crystal structure was formed on S-200 (Fig. 6f) in the fully discharged state. In contrast to the layered configuration in the interior (Fig. 6g), the outmost region appears to be a LiMn_2_O_4_-type spinel structure with a three-dimensional framework constructed by MO_6_ octahedra (Fig. 6i, j). Note that this LiMn_2_O_4_-type layer is different from the Mn_3_O_4_-type structure seen before cycling, as the TM ions are located in octahedral sites. Such a structural evolution is likely from the further rearrangements of surface atomic configurations upon charging/discharging. However, for the cycled LP electrode, the atomic configuration appears to be primarily composed of a cation-disordered rock-salt structure (Supplementary Fig. 12d–f). This difference is in agreement with the ex situ XRD measurement (Supplementary Fig. 13), which shows a well-preserved phase constitution in cycled S-200, but a much more secondary phase in the cycled LP. In addition, the cycled LP showed an amorphous feature layer with a thickness of 3–4 nm, which is more likely associated with the loss of crystallinity after accumulated oxygen loss, rearrangement of transition-metal ions, and the corrosion of the electrolyte^56–60^. For the cycled S-200 (Fig. 6f), only the outmost surface (≤1 nm) showed less ordering than that of the interior, suggesting a well-preserved crystal structure on the surface of cycled S-200. As indicated in previous studies^50,56^, the phase transition of LLO during long-term cycling follows a sequence of layered → spinel → rock salt. Therefore, we can conclude that the irreversible phase transition has been effectively suppressed in the S-deposited samples.

The anion oxidation behavior of LP and S-200 has also been investigated through ex situ XPS and X-ray absorption near-edge structure/extended X-ray absorption fine structure (XANES/EXAFS) measurements. For the XPS tests, all cycled electrodes were etched by Ar for 30 s under an etch voltage of 500 V to remove the surface species. Although the signal of lattice oxygen is influenced by the shielding effect of surface impurities in the XPS spectra, the O 1 s spectra of S-200 shows better reversible anion oxidation (well-preserved peroxo-like O_2_^2−^ peak located around 530.5 eV) than that of LP upon long-term cycling (Supplementary Figs. 14 and 15). The S *2p* spectra obtained from the S-200 electrodes in the first cycle (Supplementary Fig. 16) suggest the oxidation state of S to be SO_4_^2−^, implying the oxidation from SO_3_^2−^ to SO_4_^2−^ during the delithiation process (≤4.4 V). Within the de-/lithiation processes in the first cycle, the oxidation state of S remains at SO_4_^2−^, showing that both the S and the coordinated O ions in the outmost surface have not participated in charge compensation. After 100 cycles, even the S *2p* peak in the long-term cycled S-200 showed a lower valence state of S (∼168.5 eV), which is likely associated with the small amount of O dissolution or a side reaction between SO_4_^2−^ and the SEI/electrolyte; the oxidation state could still be represented as SO_*x*_^2−^ (3 < *x* < 4). Therefore, we can conclude that the inductive effect of the S–O bond is stable upon long-term cycling. The XAENS spectra of Mn and Ni obtained from the pristine sample (Supplementary Fig. 17) show a similar valence state of the TM ions in LP and S-200, indicating that the S adsorption has little influence to the average valence state of the TM ions. However, from the XANES spectra of Mn and Ni, and EXAFS spectra of Mn obtained from the cycled electrodes (Fig. 6k–m), both the valence state and atomic configuration vary between LP and S-200. From the Mn K-edge spectra (Fig. 6k, l), the average valence states of Mn ions in cycled LP are between Mn^2+^ and Mn^3+^ by comparing with the selected Mn references, while the S-200 shows a near +4 valence state of Mn ions. As the ideal valence state of Mn in LLO in the discharged state is +4, the low valence state of Mn in cycled LP indicates an irreversible change of Mn electronic structure upon cycling. Analogously, the Ni K-edge spectra showed a lower valence state of Ni in cycled LP, while the Ni valence state is ~+2 in cycled S-200. Therefore, it can be deduced that the transition-metal ions in cycled LP were significantly reduced, which is more likely to be associated with the irreversible processes. According to the Fourier transformed EXAFS spectra of Mn (Fig. 6m), the first coordination peak (at ∼1.3 *A*(*o*)), which is related to the Mn–O scattering path in the first shell, showed a shorter Mn–O bond in cycled LP than that in S-200, implying more significant distortion of MnO_6_ in cycled LP. On the basis of previous investigations^56,63,64^, it can be inferred that the shortened Mn–O bond in cycled LLO is either contributing to the formation of MnO_4_ tetrahedral or MnO_5_ square pyramidal ligands after the formation of oxygen vacancies in MnO_6_; both of these changes imply irreversible oxygen release upon cycling. Overall, the ex situ XPS and XAS measurements showed better-preserved anion redox as well as a diminished atomic and oxidation- state change in the S-deposited sample. The HAADF/STEM and ex situ XRD results confirm the reduced irreversible phase transition in cycled S-200. Therefore, we can then conclude that the irreversible phase transition has been effectively suppressed in the S-deposited samples.

## Discussion

DFT calculations were performed to investigate the mechanism of surface oxygen evolution in Li_2_MnO_3_ and the surface reconstruction kinetics during delithiation. The calculated LDOS and Bader charges demonstrate a preferential oxidation of O_sur_ during delithiation. This was found to be associated with the undercoordinated configuration of O_sur_ with cationic vacancies on the surface, which creates an additional O-*2p* lone-pair (lO_2*p*’_) orbital with a higher energy level than (lO_2*p*_) in O_int_. Moreover, the calculated MEP results show that the kinetically feasible O_sur_ release gives rise to surface Mn migration and thus triggers a spinel-like phase transformation. The large reverse barrier for O_2_ dissociation and Mn extraction from the spinel-like phase during discharge gives rise to the electrochemical irreversibility. To mitigate O_2_ release, we introduced less electronegative sulfur into the Li_2_MnO_3_ surface to bond with O_sur_. Our calculations show that sulfur can be stably deposited onto the surface to form oxysulfide (SO_3_)^*x*−^ and (SO_2_)^*y*−^ species, which are then further oxidized to stable polyanionic species (SO_4_)^2−^ upon delithiation. This oxidation of sulfur ions provides charge compensation on the surface in the first charge cycle, and thus prevents surface gas release. Accordingly, experimental attempts at surface sulfur deposition were carried out on Li_1.2_Ni_0.2_Mn_0.6_O_2_, and it was found that SO_*x*_ species were formed on the surface of the S-treated samples_._ The sulfur-deposited samples showed significantly reduced irreversible capacity loss in the first cycle (FCE = 82%) and superior cyclic stability (99.0% capacity and 96.0% energy density retained) upon 100 cycles at 1 C. The combination of ex situ XPS, XRD, and HAADF-STEM measurements shows a reversible anion oxidation and highly conserved crystal structure of the cycled sulfur-deposited samples, indicating the enhanced stability after sulfur deposition. The proposed surface polyanion stabilizing the surface structure can likely be generalized to materials that suffered from instability involving anion oxidation.

## Methods

### Computations

All calculations were performed with density functional theory (DFT) as implemented in the Vienna ab initio simulation package (VASP)^65^. The plane-wave basis set and the projector-augmented wave framework were used to describe the valence and core electrons. The electron exchange-correlation energy was evaluated by the Perdew–Burke–Ernzerhof (PBE) functional with a Hubbard *U* correction^66^. The effective U value with 4 eV (J = 1 eV) of Mn was adopted from previous work. The energy cutoff of the plane-wave basis set was 520 eV. The Monkhorst–Pack method with a k-point mesh of 7 × 7 × 7 and 3 × 3 × 1 for the bulk and 2 × 2 slab models calculations. Cell parameters and atomic positions were full relaxed for bulk optimization. The surface models were built after cleavage of the optimized bulk Li_2_MnO_3_ at the studied orientation. All slabs contain nine to sixteen layers of atoms, depending on different surfaces and intention, of which, the atoms in the bottom three or four layers are fixed with coordinates while other layers are fully relaxed. Sufficient vacuum thickness with 17 Å was contained in each slab to screen the interaction between the slab surfaces. Dipole correction, which is used for balancing net dipole on the surface, was considered in all slab calculations to preserve consistency. Each slab was optimized until the force per atom was less than 0.01 eV/Å.

To accurately investigate the oxygen oxidation activity, Heyd–Scuseria–Ernzerhof (HSE06) hybrid functional was applied to obtain the density of states. Strongly constrained and appropriately normed density functional (SCAN)^33^ was used to correct energy of oxygen evolution. Basin-hopping algorithm^67^, as implemented in the atomistic simulation environment^68^, was employed to search for the global minimum configurations of the slab in different delithitaion states. In total, 80 or more than 100 Li-vacancy arrangements in each delithiated state were examined. Activation barriers for Li diffusion and the reaction of O_2_ evolution and Mn migration were obtained using the climbing nudged elastic band method (cNEB)^69^. For the sulfur deposition investigation, the deposition sites on the surface were probed by placing S atom on different sites and comparing the calculated deposition energy *E*_dep._ The deposition energy *E*_dep_ was calculated as

1 $$E_{{\mathrm{dep}}} = E_{{\mathrm{slab}} \mathrm{+} {\mathrm{S}}}^{{\mathrm{hkl}}} - E_{{\mathrm{slab}}}^{{\mathrm{hkl}}} - E_{\mathrm{S}},$$

where $\it {\mathrm{E}}_{{\mathrm{slab}} \mathrm{+} {\mathrm{S}}}^{{\mathrm{hkl}}}$ is the energy of the slab with deposited S, $\it E_{{\mathrm{slab}}}^{{\mathrm{hkl}}}$ is the energy of the clean surface, and $E_{\mathrm{S}}$ is the energy of the elemental sulfur, which is referred to Materials project^70^. The gas-evolution behavior in the presence of solvent was investigated using an implicit solvent model as implemented in the VASPsol package^38^. The reaction between surface and ethylene carbonate (EC) molecule was explored through EC ring-opening reaction after adsorption on delithiated surface. The reaction energy Δ*E* was calculated as

2 $$\Delta E = E_{{\mathrm{slab}}+{\mathrm{EC}}} - E_{{\mathrm{slab}}} - E_{{\mathrm{EC}}},$$

where *E*_slab+EC_ is the energy of the slab after EC molecule adsorption, *E*_slab_ is the energy of the surface before EC adsorption, and *E*_EC_ is the energy of EC molecule. All energies were corrected by the SCAN functional.

### Synthesis of Li_1.2_Ni_0.2_Mn_0.6_O_2_

Li_1.2_Ni_0.2_Mn_0.6_O_2_ was synthesized through co-precipitation method. Lithium hydroxide monohydrate (LiOH·H_2_O), manganese sulfate monohydrate (MnSO_4_·H_2_O), nickel sulfate hexahydrate (NiSO_4_·6H_2_O), and sodium carbonate (Na_2_CO_3_) were purchased from Sigma-Aldrich and used without further purification. A 0.8 M aqueous solution of transition metals was obtained by dissolving MnSO_4_·H_2_O and NiSO_4_·6H_2_O with the Mn:Ni molar ratio of 3:1 into 10 ml of deionized water. In total, 1 g of Na_2_CO_3_ was added into the mixture of 10 ml of ethanol and 20 ml of deionized water to form the precipitating agent; the solution was stirred until Na_2_CO_3_ was completely dissolved. The precipitating agent was added into the solution of transition metals drop by drop under continuous stirring; the solution was kept with stirring for 1 h and then washed by deionized water and ethanol before drying at 80 °C. The obtained precursor was mixed with LiOH with a molar ratio of Li:M (M = Mn + Ni) = 1.65:1 and then heated to 800 °C for 12 h with a ramping rate of 3.5 °C/min. The products were washed with deionized water and ethanol and finally dried in an oven at 60 °C for 4 h. The obtained pristine Li_1.2_Ni_0.2_Mn_0.6_O_2_ powders were labeled as LP.

### Deposition of sulfur

In total, 40 mg of as-prepared Li_1.2_Ni_0.2_Mn_0.6_O_2_ powder and different amount of sulfur powder (Aldrich) were placed in two crucibles separately before transferring into tube furnace; the tube was evacuated and sealed to avoid the oxidation of sulfur. To create the sulfur atmosphere and promote the sulfur absorption, the furnace was heated to 250 °C with the ramping rate of 15 °C/min and maintained for 20 min until naturally cooled. Further vacuum annealing was carried out for the obtained products at 250 °C for 20 min to remove the residual elemental sulfide. Different amount of sulfide was used to make sulfur atmosphere with various concentrations; samples exposed in sulfur atmosphere with the sulfur resource of 50, 200, and 500 mg were labeled as S-50, S-200, and S-500, respectively. All the S-deposited samples were stored into Ar-filled glovebox directly after the S deposition.

### Characterization

The phase constitution was investigated by XRD patterns that were performed on PANalytical–Empyrean XRD with Cu K*α* radiation (*λ* = 0.15405 nm, 40 kV, 40 mA). SEM observation was carried out on a FEI Sirion XL30 SEM.

### HAADF-STEM

For the preparation of HAADF-STEM samples before cycling, samples were first grinded in a mortar and then dispersed in ethanol, the solution was sonicated for 30 min, and the supernatant was deposited onto holey carbon grids. For the preparation of HAADF-STEM samples after cycling, all the processes were handled in Ar-filled glovebox. The cycled batteries were dissembled, and the electrodes were first immersed in dimethyl carbonate for 24 h; further rinses were carried out by washing the electrodes with dimethyl carbonate five times to remove the residual electrolytes. Active materials were scraped from the electrodes and crushed in the mortar in anhydrous hexane before depositing onto holey carbon grids. The specimens were sealed in Ar-filled container and stored in a glovebox until transferring into a microscope column. The HAADF-STEM images were obtained on a double-aberration-corrected S/TEM (FEI Titan Cubed G2 60-300) operated at 300 kV.

### X-ray spectroscopy characterization

XPS measurements were performed on a Thermo Fisher Scientific ESCALab 250Xi. The cycled batteries were dissembled and washed in a glovebox; the electrodes were immersed in dimethyl carbonate for 24 h and further washed by dimethyl carbonate five times to remove the residual electrolytes. The samples were dispersed onto the sample holders and then sealed into a homemade protector inside a glovebox, and the protector was stored in the glovebox before transferring into the XPS chamber. For the ex situ XPS measurements, to eliminate the shielding effect of residual electrolyte and solid electrolyte interphase (SEI) layer on the surface, all electrodes were etched by Ar for 30 s under an etch voltage of 500 V. The calibration of all the spectra is carried out with the C 1 s peak position at 284.8 eV. Each fitting was carried out with a minimum number of components involved, and error bars were calculated by adopting the method in ref. ^20^, which probes the XPS detection limitation by fitting pristine samples with three and four peaks. XANES and EXAFS were carried out at the Canadian Light Source Inc.; all XAS data were processed using Athena program.

### Electrochemical characterization

The samples were mixed with Super P and poly(vinylidene fluoride) (PVDF) with the weight ratio of 7.5:1.5:1. N-methyl-2 pyrrolidone (NMP) was added into the mixture until a uniform slurry was formed; the slurry was spread onto Al foil and dried at 60 °C. The foil was punched into square pieces with the typical loading of active materials ranging from 1.0 to 2.0 mg cm^−2^. The electrodes were further dried in a vacuum oven at 120 °C for 10 h before transferring into the glovebox. The electrochemical measurements were performed with coin cells (CR2025), in which lithium foil acted as both counter- and reference electrodes. The electrolyte (CAPCHEM) was composed of 1 M LiPF_6_ solution in ethylene carbonate (EC)/dimethyl carbonate (DMC) mixture solution (1:1 by volume), and the Celgard 2400 membrane was used as separator. Galvanostatic charge/discharge cycling was tested on CT2001A battery test systems (LAND Wuhan Corp., China) within the voltage range of 2.0–4.8 V at room temperature.

## Supplementary information

Supplementary Information

## Data Availability

The data that support the findings of this study are available from the corresponding author upon reasonable request.
