# Supplementary material for: Highly reversible oxygen redox in layered compounds enabled by surface polyanions
Source: Nat Commun. 2020 Jul 8;11:3411. doi: 10.1038/s41467-020-17126-3 (PMC7343805; doi:10.1038/s41467-020-17126-3)
Supplement: Supplementary file 1 — Supplementary Information [file 41467_2020_17126_MOESM1_ESM.pdf]

Supplementary Information

Highly reversible oxygen redox in layered compounds enabled by surface polyanions

by Qing Chen *et al*.

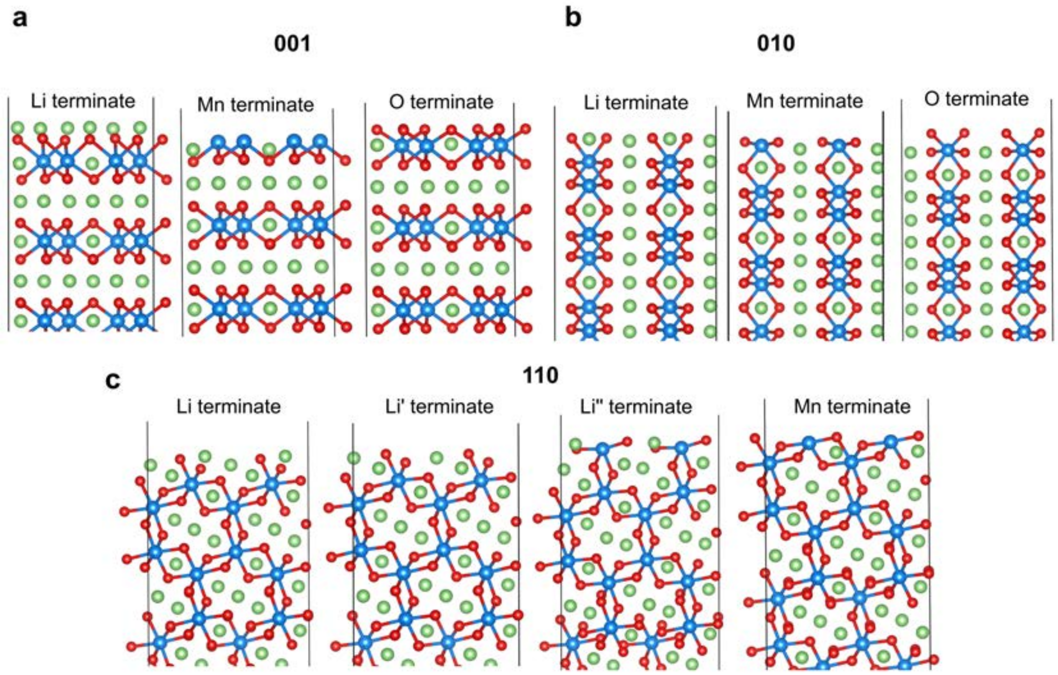

Supplementary Figure 1 | Three low index surfaces of Li_2MnO_3 with different terminates. a, (001) surface. **b,** (010) surface. **c,** (110) surface. The blue, green, and red color respectively represent Mn, Li, and O ions. Li- terminate, Mn- terminate, and O-terminate are considered in (001) and (010) slab, three Li- terminates and Mn- terminate are considered in (110) slab. All surfaces with Li-terminate are calculated to have lower energy than with other terminations. Delithiation potential of the outermost Li in each slab is also combined to evaluate the stability, and calculated to be 2.8V, 4.4V and 3.7V in (001), (010) and (110) slab, respectively. The delithiation potential with 2.8 V in (001) surface is below the open-circuit voltage (OCV) of the system, indicating the exposed Li layer will dissolve in the electrolyte when impregnation with electrolyte, whereas the covered Li in (010) and (110) slab are stable until charging.

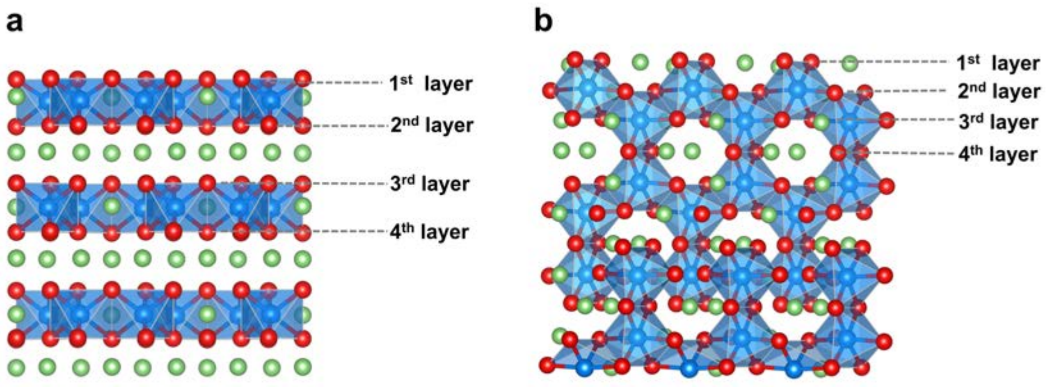

Supplementary Figure 2 | Surface structure of Li_2MnO_3 and the illustration of surface oxygen from the first layer to the fourth layer. a, (001) surface. **b,** (110) surface.

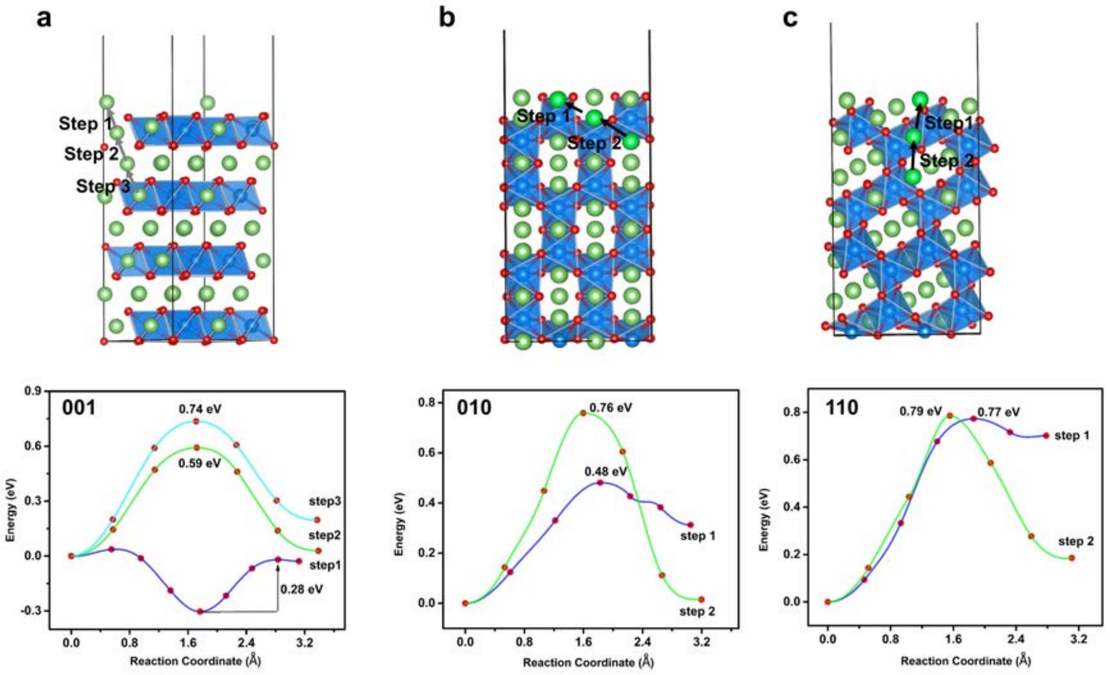

Supplementary Figure 3 | Li ion diffusion paths in different surface of Li_2MnO_3 and the corresponding migration barrier. a, (001) surface. **b,** (010) surface. **c,** (110) surface. Li vacancy always starts to appear in the outmost layer when Li extraction is beginning. The first step of extraction internal Li is sublayer Li diffusion to outlayer, as labeled with step 1; the next step is intimal layer Li diffusion to sublayer, labeled as step 2. The calculated migration barrier of each step shows that Li mobility in (001) surface is not more difficult than that in intra-layer, (010) and (110) surfaces, which is the unanimous Li ion conductive pathway. Thus, the three studied surfaces are electrochemical active at least in initial stage of delithiation.

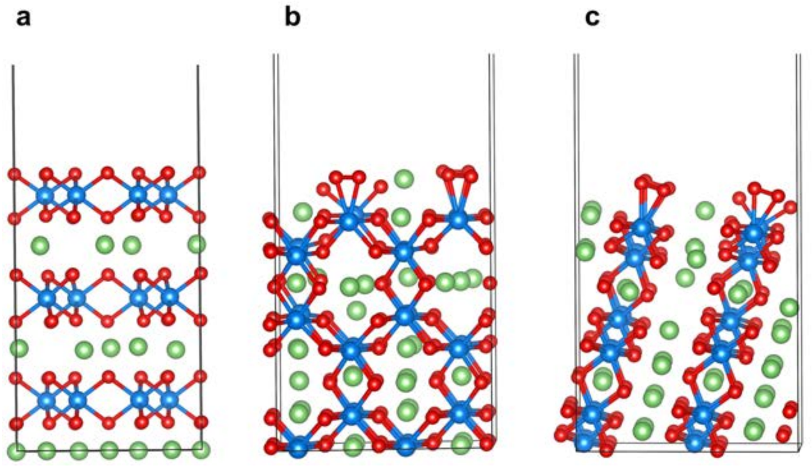

Supplementary Figure 4 | Delithitated structures of three surfaces in Li_2MnO_3 with 37.5% Li extraction. a, (001) surface. **b,** (010) surface. **c,** (110) surface. Li vacancy is primarily distributing in the outer part of the slabs. Three pairs oxygen ions in the outmost layer of (010) and (110) surfaces rotated to form O-O bonding spontaneously upon delithiation.

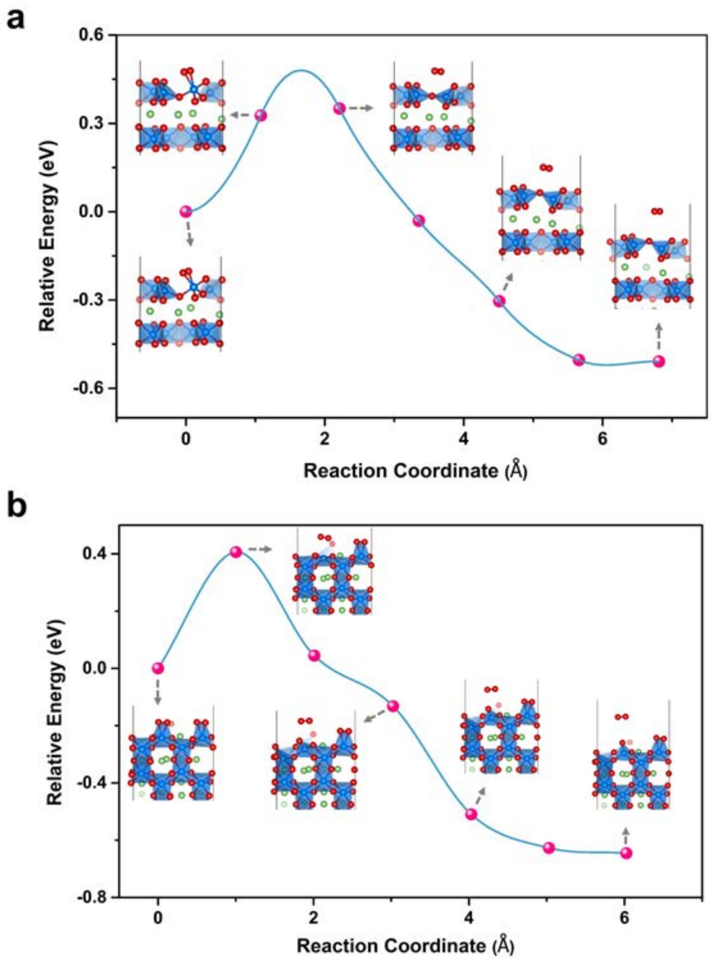

Supplementary Figure 5 | Strongly constrained and appropriately normed density functional (SCAN) calculated MEP plot for O_2 evolution at the surface of Li_2MnO_3. a, (001) surface. **b,** (010) surface.

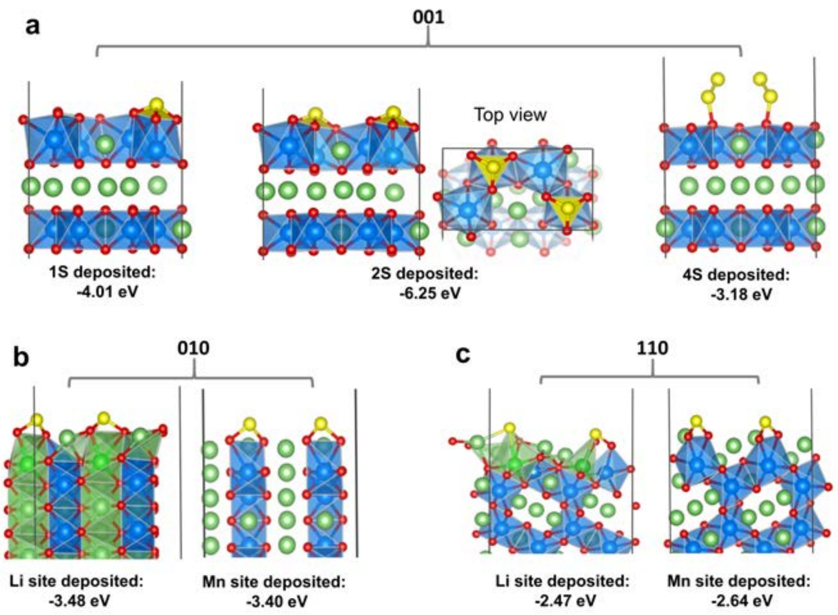

Supplementary Figure 6 | Sulfur depositional surface structures and the calculated deposition energy. a, Structures of different amount S deposited on (001) surfaces and the calculated deposition energy, **b, c,** Structures and the calculated deposition energy of S deposited on Li ion and Mn ion of (010) and (110) surface. For (001) slab, since four equivalent Mn atoms lies in the outmost layer, various amount of S atoms were placed on the slab, to find that when deposition of one and two S atoms, a more stable sulfite-like species (SO_3^{2-}) is formed with the deposition energy of -4.01 eV and -6.25eV. Whereas less stable S-S bond, with the deposition energy of -3.18 eV, is generated when deposition of four S atoms. This indicates that sulfur deposition on (001) slab is an exothermic reaction, besides, comparing to large quantity S, moderate S deposition seems to facilitate a compact and stronger binding with the surface. For (010) and (110) slab, two possible deposition sites-on the top of Li and Mn at the outmost layer-were examined, to show that each site deposition is exothermic, and the preferred deposition site is top of Li in (010) surface and top of Mn in (110) surface. Distinguished form (001) surface, a $(SO_2)^{x-}$ species is generated on both surfaces. Such generated (SO_3^{2-}) and $(SO_2)^{x-}$ species are calculated to be stable, that is, unlikely decomposed to any other molecular and desorbed from the surface.

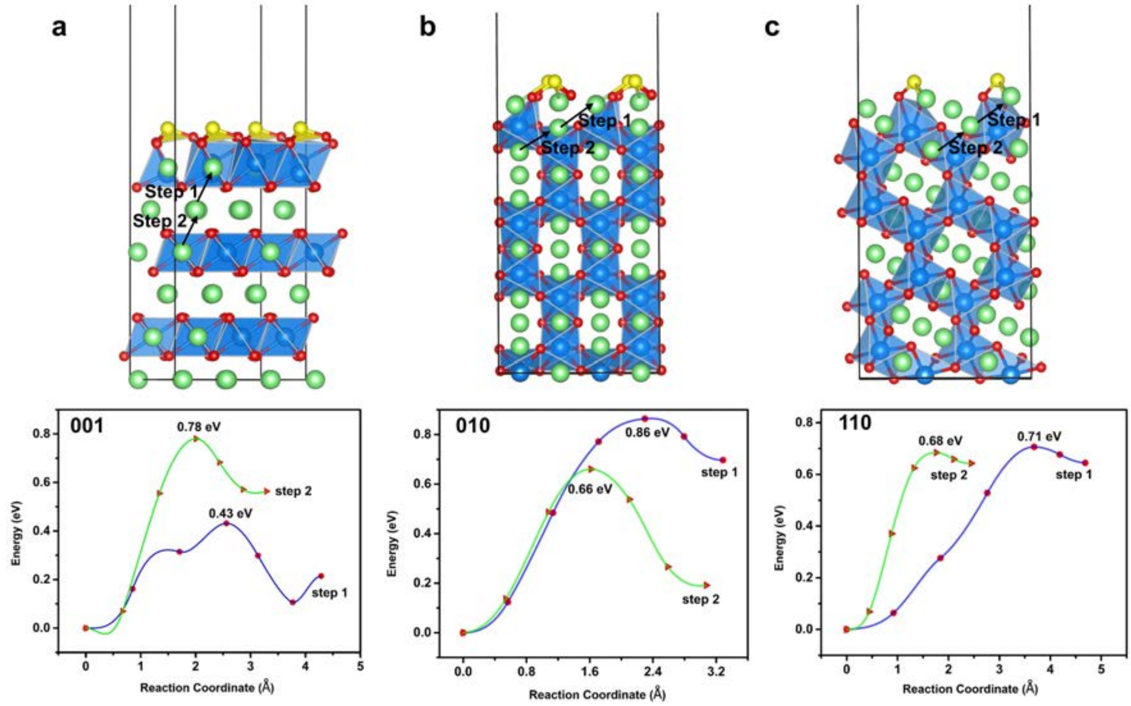

Supplementary Figure 7 | Li ion diffusion paths in S deposited surfaces of Li_2MnO_3 and the corresponding migration barriers. a, (001) surface. **b,** (010) surface. **c,** (110) surface. The calculated barrier of each step, comparing with the original surface (Supplementary Figure 3), indicates that Li extraction from surface of Li_2MnO_3 does not kinetically hindered by sulfur deposition.

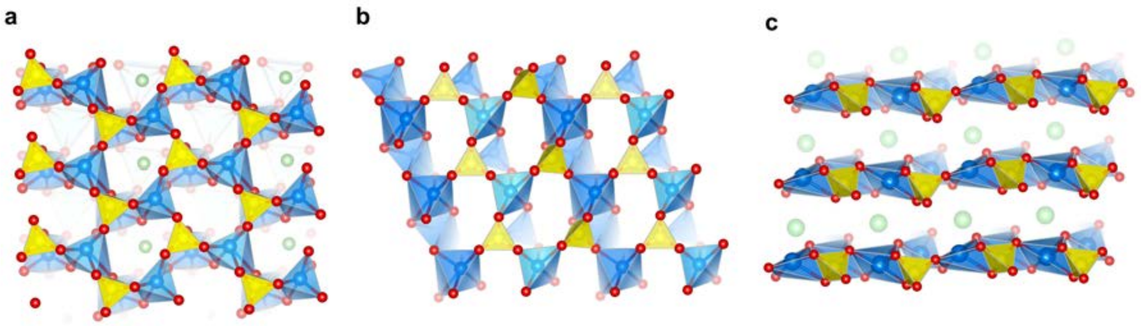

Supplementary Figure 8 | Top view of sulfur depositional surface structures upon 37.5% delithiation. a, (001) surface. **b,** (010) surface. **c,** (110) surface. Each exposed O ion in three surfaces bonds to deposited S ion and constitutes stable sulfate species SO_4^{2-}.

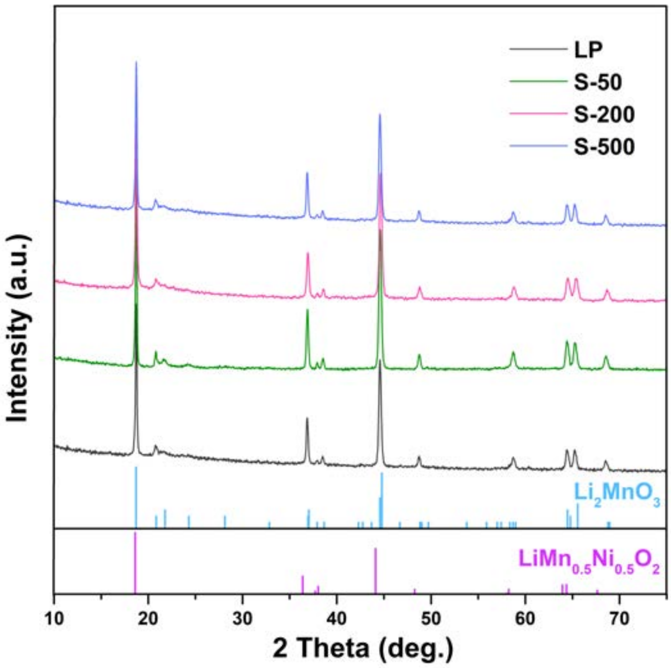

Supplementary Figure 9 | Powder XRD patterns of products before and after S absorption. The standard Bragg position of Li_2MnO_3 and $LiMn_{0.5}Ni_{0.5}O_2$ are indicated as cyan and purple vertical tick marks, respectively.

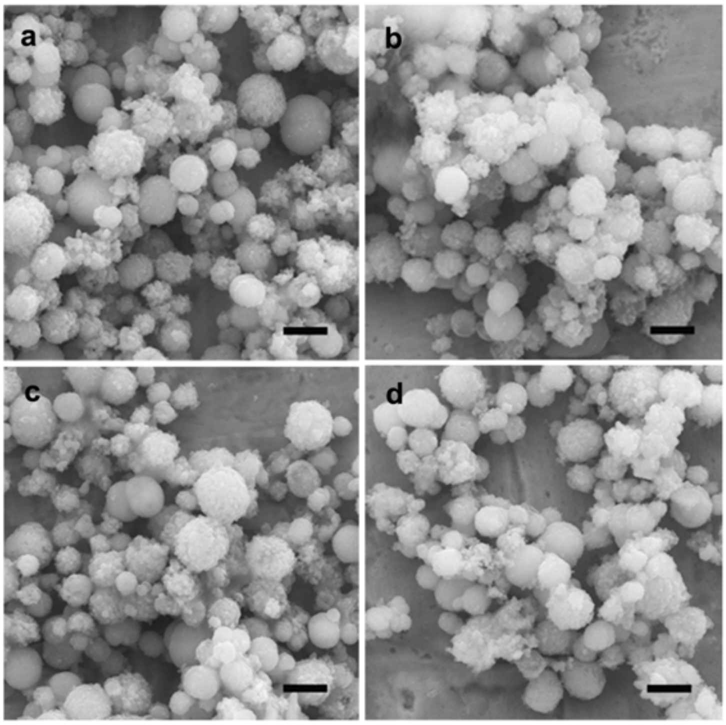

Supplementary Figure 10 | SEM images of products before and after S absorption. a, LP, **b,** S-50, **c,** S-200, **d,** S-500. Scale bar denote 2 μm.

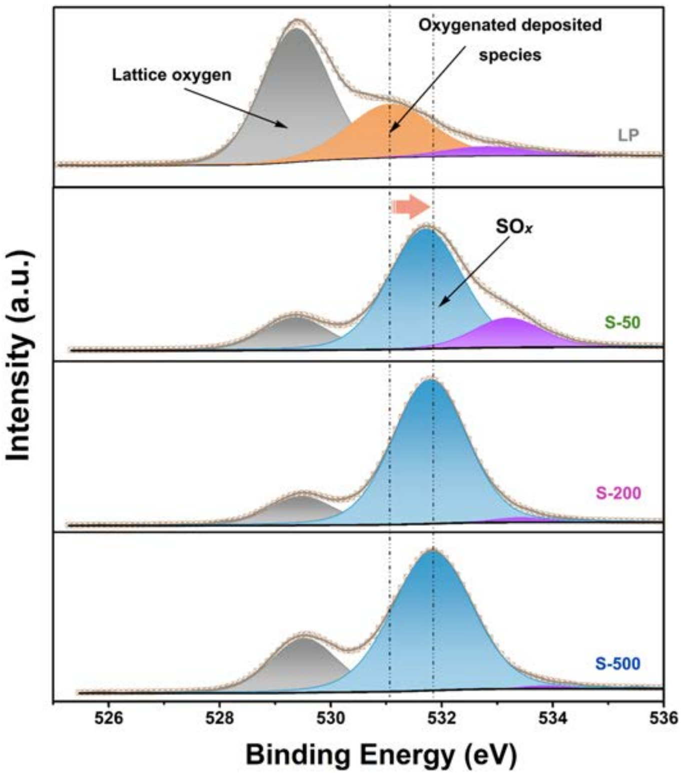

Supplementary Figure 11 | O 1*s* spectra of products before and after S absorption. The differentiated and imitated peaks are shown in different colors. The grey peaks located near 529.5 eV are attributed to the lattice oxygen (M-O) in transition metal oxides; the orange and cyan peaks located near 531 eV and 532 eV corresponding to the oxygenated deposited species and SO_x species, respectively.

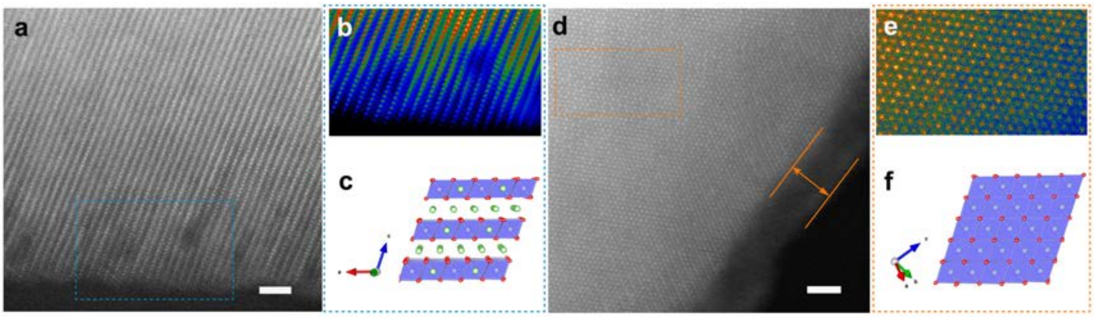

Supplementary Figure 12 | The atomic structural evolution of LP before and after cycling. a-c Atomic configuration of the LP before cycling. The overview HAADF-STEM image (**a)** shows the crystal structure from surface to the bulk of LP. The surface region marked by the blue dashed square in **(a)** has been FFT-filtered and shown in **(b)**, and the corresponded atomic model is shown in **(c).** The spots with higher contrast in **(b)** presented TM ions with higher Z than Li ions, the Li, M, and O ions has been showed as green, blue and red balls in **(c)**, respectively. **d-f** Atomic configuration of the LP electrode after 100 cycles. The HAADF-STEM image (**d**) shows the rock-salt feature in both the surface and interior areas. The atomic configuration, marked by the orange dashed square in (d), has been FFT-filtered and shown in (e), and the corresponded atomic model is shown in (f). The amorphous surface region in cycled LP was marked by orange lines. Scale bar denote 2 nm.

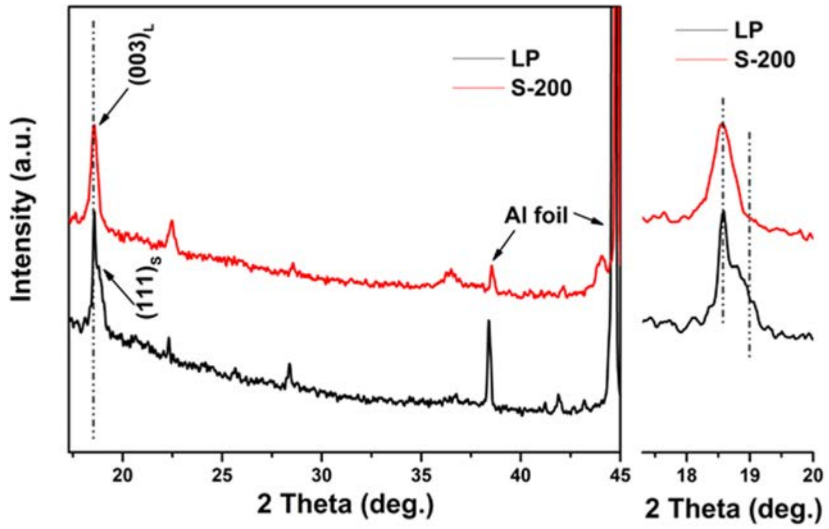

Supplementary Figure 13 | *Ex-situ* XRD pattern of LP and S-200 after 100 cycles under 0.5C.

The peaks marked as $(003)_L$ referred to the reflection of (003) plane of layered phase, and the peaks marked as (111)s located at higher angle referred to the reflection of (111) plane of spinel phase. As the phase transition of LLO during long-term cycling follow a sequence of layered→spinel→rock-salt, we can then conclude that less phase transition existed during the cycling of S-200. Noted that even large amount of rock-salt phase has been observed in HAADF/STEM, the content of rock-slat phase could not be deduced from *ex-situ* XRD pattern as the reflections referred to rock-salt phase has been overlapped with that of Al foil (around 38° and 45°).

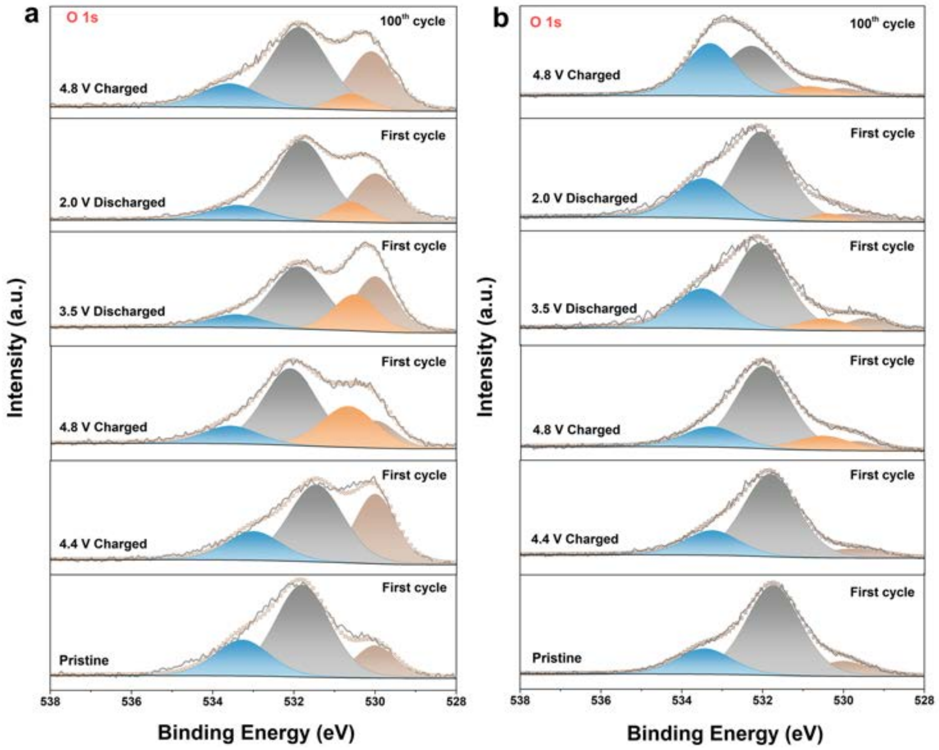

Supplementary Figure 14 | Changes in oxygen oxidation states of LP and S-200 during the cycling. a, O 1*s* spectra of LP at various charge states during cycling. **b,** O 1*s* spectra of S-200 at various charge states during cycling. Four kinds of O oxidation states are assumed to exist in the products based on previous researches,[1,2] which are: lattice oxygen (529.5-530.0 eV); peroxo-like O_2^{2-} (~530.5 eV); oxygenated deposited species (531.5-532.5 eV); electrolyte oxidation (533~534 eV). The fitting was carried out with minimum peaks involved for each sample, the full-widths at half-maximum (FWHMs) are restricted to be 1.2-1.6 eV and the L/G ratio was fixed as 20%. The O_2^{2-} feature peak are relative lower in the O 1*s* spectra of S-200, which may be influenced by the strong shielding effect of impurities on the surface. However, attempts to resolve the O1s spectra without that peak resulted in unreasonably FWHMs (>2 eV) and poor fits.

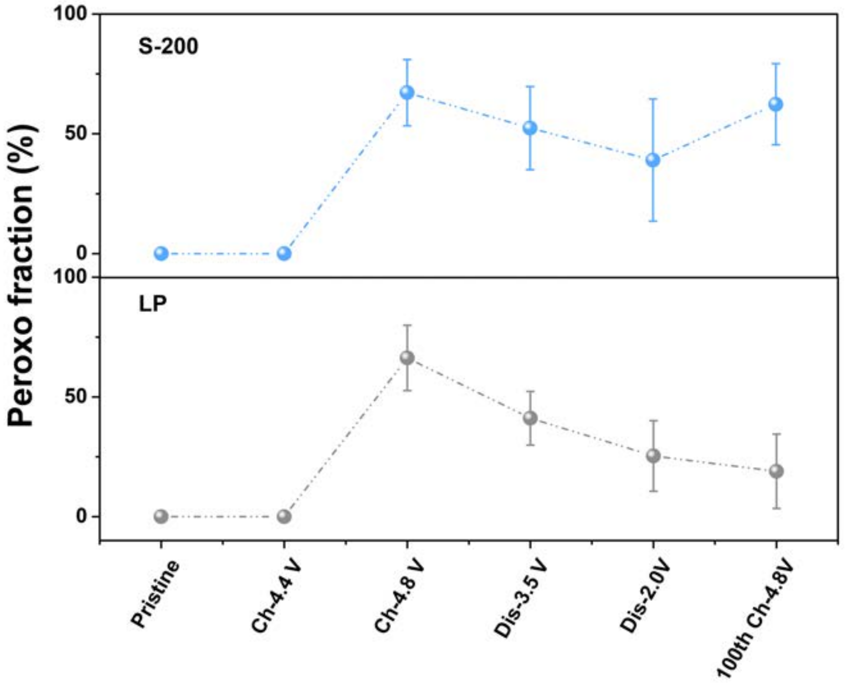

Supplementary Figure 15 | Fraction of peroxo species (O_2^{2-}) within the lattice oxygen (O^{2-} and O_2^{2-}) under different voltages. Each fitting was carried out with a minimum number of components involved, and error bars were calculated by adopting the method in ref. 1, which probes the XPS detection limitation by fitting pristine samples with three and four peaks.

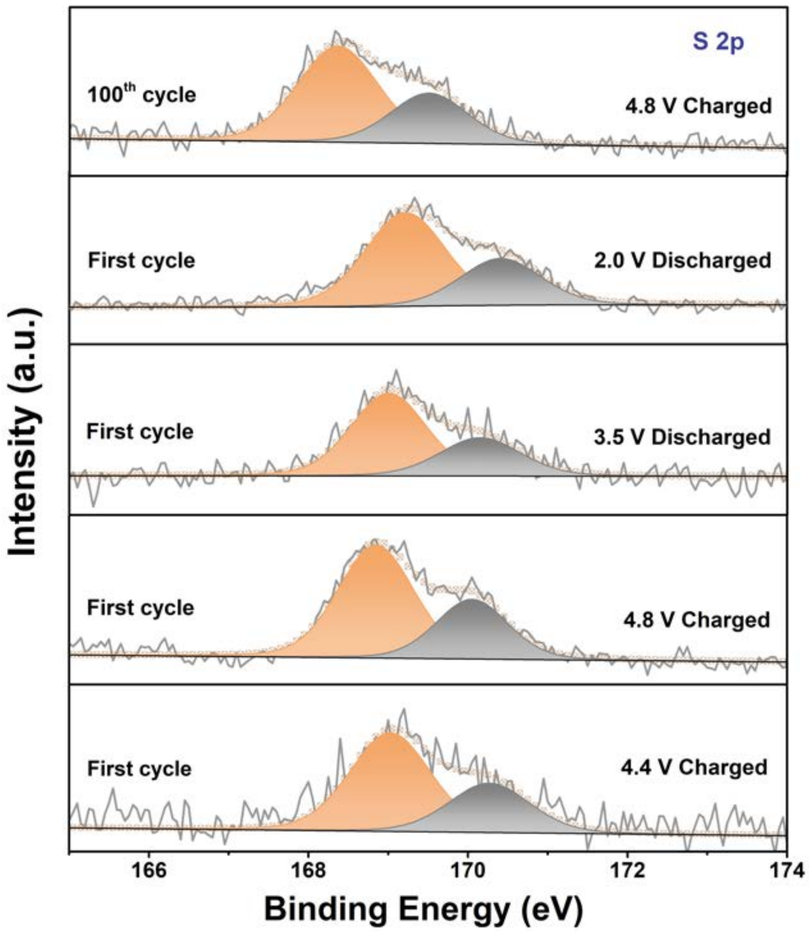

Supplementary Figure 16 | Changes in sulfur oxidation states of S-200 during the cycling. The differentiation and imitation suggested the oxidation state of S to be SO_4^{2-} (S 2$p_{3/2}$ located near 169 eV and S 2$p_{1/2}$ located near 170.2 eV) in the first cycle, which is in accordance with our DFT calculations. However, the shift of S peaks in the 100th cycle indicated that SO_4^{2-} group has been reduced during long-term cycling, suggesting side reactions may occurred between SO_4^{2-} group and electrolyte after repetitive charge-discharge.

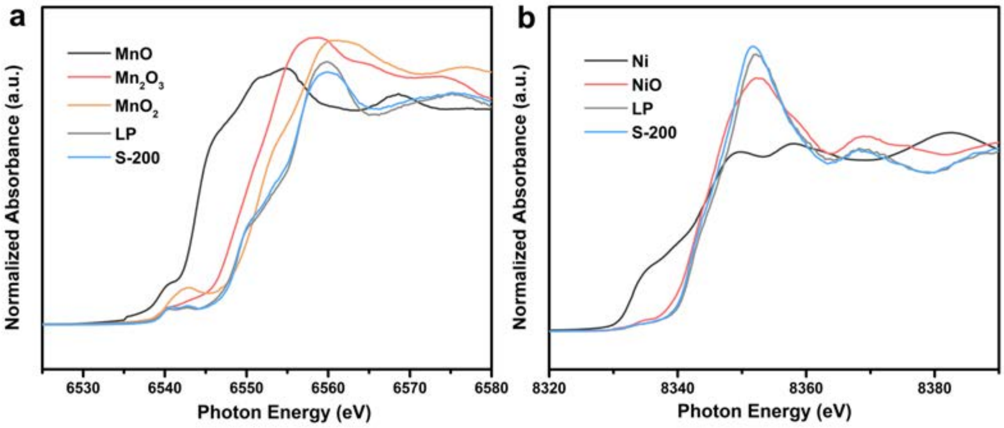

Supplementary Figure 17 | Oxidation states of LLO before and after S adsorption. a, Normalized Mn K edge XANES spectra of pristine LP and S-200. The valence states of Mn were determined by comparison against to the spectra of pure-phase MnO, Mn_2O_3 and MnO_2. **b,** Normalized Ni K edge XANES spectra of pristine LP and S-200. The valence states of Ni were determined by comparison against to the spectra of pure-phase Ni and NiO.

Supplementary References:

1. McCalla, E. et al. Visualization of O-O peroxo-like dimers in high-capacity layered oxides for Li-ion batteries. *Science*. **350** 1516-1521 (2015).
2. Wilde, L. et al. Adsorption and temperature-dependent decomposition of SO_2 on Ni(110): an XPS and XAFS study. *Surf. Sci*. **405**, 215–227 (1998)).
